# Assessment of clinically related outcomes and biomarker analysis for translational integration in colorectal cancer (ACROBATICC): study protocol for a population-based, consecutive cohort of surgically treated colorectal cancers and resected colorectal liver metastasis

**DOI:** 10.1186/s12967-016-0951-4

**Published:** 2016-06-29

**Authors:** Kjetil Søreide, Martin M. Watson, Dordi Lea, Oddmund Nordgård, Jon Arne Søreide, Hanne R. Hagland, ACROBATICC collaborators

**Affiliations:** Department of Gastrointestinal Surgery, Stavanger University Hospital, POB 8100, 4068 Stavanger, Norway; Gastrointestinal Translational Research Unit, Laboratory for Molecular Biology, Stavanger University Hospital, Stavanger, Norway; Department of Clinical Medicine, University of Bergen, Bergen, Norway; Department of Pathology, Stavanger University Hospital, Stavanger, Norway; Department of Haematology and Oncology, Stavanger University Hospital, Stavanger, Norway; Centre of Organelle Research (CORE), University of Stavanger, Stavanger, Norway

**Keywords:** Biomarker, Cancer, Population-based, Translational research, Colorectal cancer, Liver metastasis, Circulating tumour cells, Genetics

## Abstract

**Background:**

More accurate predictive and prognostic biomarkers for patients with colorectal cancer (CRC) primaries or colorectal liver metastasis (CLM) are needed. Outside clinical trials, the translational integration of emerging pathways and novel techniques should facilitate exploration of biomarkers for improved staging and prognosis.

**Methods:**

An observational study exploring predictive and prognostic biomarkers in a population-based, consecutive cohort of surgically treated colorectal cancers and resected colorectal liver metastases. Long-term outcomes will be cancer-specific survival, recurrence-free survival and overall survival at 5 years from diagnosis. Beyond routine clinicopathological and anthropometric characteristics and laboratory and biochemistry results, the project allows for additional blood samples and fresh-frozen tumour and normal tissue for investigation of circulating tumour cells (CTCs) and novel biomarkers (e.g. immune cells, microRNAs etc.). Tumour specimens will be investigated by immunohistochemistry in full slides. Extracted DNA/RNA will be analysed for genomic markers using specific PCR techniques and next-generation sequencing (NGS) panels. Flow cytometry will be used to characterise biomarkers in blood. Collaboration is open and welcomed, with particular interest in mutual opportunities for validation studies.

**Status and perspectives:**

The project is ongoing and recruiting at an expected rate of 120–150 patients per year, since January 2013. A project on circulating tumour cells (CTCs) has commenced, with analysis being prepared. Investigating molecular classes beyond the TNM staging is under way, including characteristics of microsatellite instability (MSI) and elevated microsatellite alterations in selected tetranucleotides (EMAST). Hot spot panels for known mutations in CRC are being investigated using NGS. Immune-cell characteristics are being performed by IHC and flow cytometry in tumour and peripheral blood samples. The project has ethical approval (REK Helse Vest, #2012/742), is financially supported with a Ph.D.-Grant (EMAST project; Folke Hermansen Cancer Fund) and a CTC-project (Norwegian Research Council; O. Nordgård). The ACROBATICC clinical and molecular biobank repository will serve as a long-term source for novel exploratory analysis and invite collaborators for mutual validation of promising biomarker results. The project aims to generate results that can help better discern prognostic groups in stage II/III cancers; explore prognostic and predictive biomarkers, and help detail the biology of colorectal liver metastasis for better patient selection and tailored treatment. The project is registered at http://www.ClinicalTrials.gov NCT01762813.

## Background

Colorectal cancer represents a formidable health burden worldwide with an expected 60 % increase towards 2030 [1]. Currently, CRC ranges as the second most frequent cancer in both genders in the Western world. Despite an increasingly favourable prognosis due to stepwise progression in surgical and oncological management [2], still about 40–50 % will develop metastasis and die from the disease. The liver is the most frequent site for metastasis, followed by the lungs, and is also the rate-limiting organ step for long-term survival. For non-metastatic disease, prognosis is guided through the tumour-node-metastasis (TNM)-system, which heavily relies on the status of lymph nodes for current staging [3, 4]. Further, node status may vary with the underlying molecular composition of primary tumour [5, 6]. Also, more refined node-examination including ultrastaging by immunohistochemistry, sentinel node techniques or use of molecular markers to identify malignant cells have not yielded a higher precision overall [7, 8]. Furthermore, other methods and techniques of staging patients, such as the use of “liquid biopsies” i.e. by investigating circulating tumour cells (CTCs) or other tumour constituents in peripheral blood (e.g. microRNAs), may prove to have higher prognostic and predictive value in both primary and metastatic CRC [9–12]. Notably, well-described molecular routes of progression in CRC have been linked to specific prognosis and outcomes, including microsatellite instability (MSI), CpG-island methylator phenotype (CIMP) and chromosomal instability (CIN) [13–17].

**Fig. 1:**
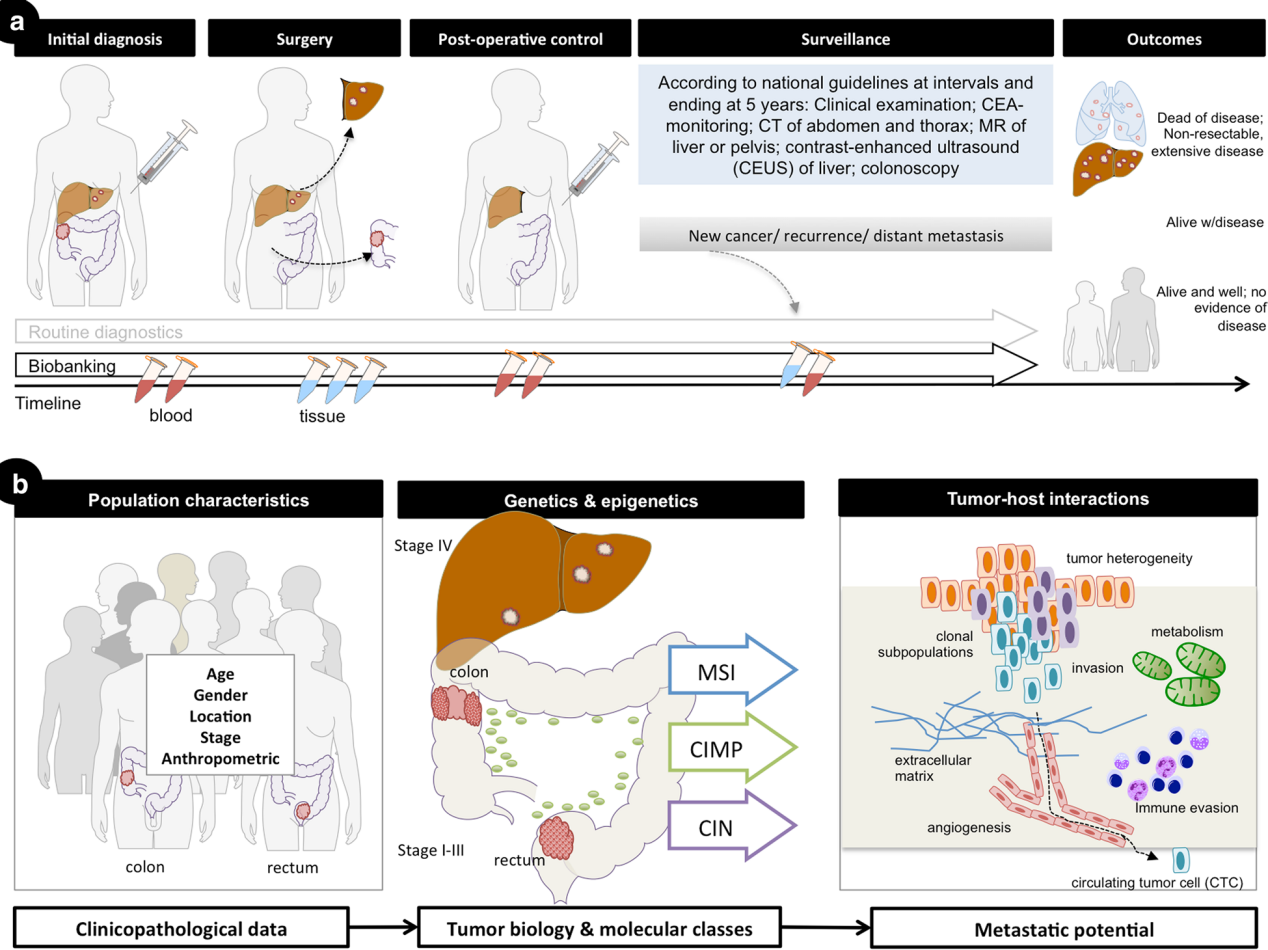
The ACROBATICC project flow sequence and rationale for cancer biology investigation. **a** Illustrated is a simple workflow of patients’ recruitment and samples of blood (*red vials*) and tissues (*blue vials*) from initial diagnosis, before and after surgery and during follow up. Overall, disease-free and cancer-specific survival will be analysed at 5 years. **b** Illustrated are the specific levels of patient information gathered for prognostic and predictive use, ranging from clinicopathological characteristics (such as sex, age, body weight and height) to genetic and epigenetic mechanisms (including microsatellite instability; CpG-island methylator phenotypes and chromosomal instability) and specific tumour-host interactions (such as immune-response in tumor; cancer metabolism and role of circulating tumor cells). With access to newer techniques and development of novel hypothesis, the project will allow for exploration of other predictors, as well as serve as external validation cohort in collaborative research

While the TNM is the best staging system at hand for clinical decision making, the TNM system is known to be imperfect [4], and substantial over- and undertreatment results from failure to accurately predict disease outcomes. Indeed, increased knowledge of cancer heterogeneity has led researchers and clinicians alike to pursue better ways of stratifying therapy to individual risk and effects response and efficacy of therapy [18]. One suggested consensus taxonomy has emerged for novel risk-groups [19], however these have yet to be implemented in clinical practice. Variation in definition of common denominators for disease stratification may be due to a number of reasons, including heterogeneous patient groups investigated; investigations done on patients recruited to randomized trial with strict inclusion criteria; variation in tumour sampling, and; variation in molecular analyses and techniques, to mention but a few [14, 20–22]. Thus, exploring biomarkers and the described genetic and epigenetic pathways in CRC [23, 24] by well-defined population cohorts with access to biobanking beyond routine samples is crucial (Fig. 1).

The aim of this prospective project is to facilitate prospective accrual of patients with snap-frozen blood samples and fresh-frozen tissue outside routine clinical care, for more refined molecular evaluation (Fig. 1). For one, we will explore the role of microsatellite instability (MSI) and specifically a form found in tetranucleotide repeats (elevated microsatellite alterations in selected tetranucleotides; EMAST), which is found at varying frequencies in several cancers [25]. EMAST is a less-well described molecular trait in CRC, however, recent data point to a prognostic role and as a potential modulator of cancer biology [26–28]. The role of MSI and EMAST in relation to presently well-described prognostic mutations, such as KRAS and BRAF [29, 30], is not well described. Also, investigation into putative mechanisms leading to EMAST and the consequence for a predictive and prognostic role is warranted. Second, a cohort of consecutive patients will be evaluated for CTCs in resectable stage I–III CRC for its prognostic value. Third, as the role of the immune system is increasingly recognized as an integral component to carcinogenesis, cancer biology and patient prognosis [31–33], the project will investigate elements of the immune system in tumour samples as well as in peripheral blood. Finally, additional expansion of related projects are emerging with novel techniques, particularly with the availability of next-generation sequencing. Here we present the study design and protocol for a population-based translational cancer research project.

## Methods

### Study ethics approval

The project and research biobank has been approved by the Regional Ethics Committee of the Western Health Authority (REK Helse Vest, #2012/742) and by the Institutional review board (Helse Stavanger HF Protocol Record #29034/2012). The project has been registered at www.ClinicalTrials.gov NCT#01762813.

### Consenting

Patients are informed and consented in the surgery outpatient clinic or, if directly admitted, in the surgical ward, before admission for surgery. A trained research nurse certified in Good Clinical Practice does consenting of patients and registration.

### Study population

Stavanger University Hospital serves as the only hospital for a population of about 350,000 inhabitants in the South-Western part of Norway. The population is predominant of Caucasian origin, the average population age slightly younger than the national average. Socioeconomic differences are not extreme in the country; the life expectancy is just below 80 years for men and about 81 years for women. With no other competing hospitals in the region and a social security system ensuring equal care for all patients, the study population allows for reliable, unselected, population-based and representative data sampling with little risk of bias.

We have previously reported epidemiological characteristics to other disease categories based on the same non-selected, unbiased conditions, which should validate the methodology to the current population-based perspective [34–39].

With low migration in the region, long-term follow up is feasible and allows for high precision in catching new events (disease related or other) with impact on outcomes, as previously reported [40, 41]. The risk of loosing patients to long-term follow-up is thus minimal.

All Norwegian citizens have a unique 11-digit social security number that allows identification through the hospital electronic hospital records with national registries, including the Norwegian Patient Register and the Cancer Registry of Norway. All CRC patients are registered to the National Colorectal Cancer Registry via an electronic template record form. The study will comply to the strengthening the reporting of observational studies in epidemiology (STROBE) recommendations on what should be included in an accurate and complete report of any observational study [42].

### Inclusion and exclusion criteria

All consecutive patients diagnosed with operable primary or metastatic colorectal cancer and able to provide informed, written consent are included. Participants can at any time withdraw from the study without need for providing any explanation for the withdrawal, upon which the records will be destroyed and deleted. Excluded are patients presenting as emergencies and unable to consent, or patients unable to understand oral and written Norwegian or, patients whose cognitive status does not allow for informed consent.

### Study period

The study commenced in January 2013 and recruitment is ongoing, for an expected closure of recruitment in 2018, with final 5-year follow-up to be completed in 2022.

### Study number and sample size

For prognostic information and estimated sufficient numbers of patients and events, we expect to recruit about 150 patients per year. With an expected 35–45 % recurrence rate within 5 years of primary diagnosis—which is expected from previous regional and national data of curatively resected CRC in Norway [2, 40, 43]—and, an accrual commencing over at least 5 years (2013–2018) we expect to have about 750 patients with operable primary and/or metastatic CRC for evaluation at the end of the period. In stage I-III CRC, the recurrence rate at 35–45 % by 5 years [2, 40, 43] should yield appropriate number of events (cancer-specific survival) for creation of test-sets, validation-sets and prospective evaluation. Recurrence rates are expected to be even higher in colorectal liver metastasis (>80 % recurrence within 5 years), suggesting fewer patients are needed to evaluate the endpoint.

### Clinical work-up and care

The Department of Gastrointestinal Surgery provide all clinical work-up and surgical care for patients with colorectal cancer and subsequent evaluation for metastases, resectable or non-resectable. Oncologic care is provided at the Department of Oncology. All radiologic work-up (except PET/CT scans) are performed at the Department of Radiology. Specimen evaluation and tissue blocks preparation for routine diagnostics are performed at the Department of Pathology. Routine blood tests are analysed at the Department of Clinical Chemistry. Storage of research samples (fresh-frozen) are archived in an intramural research biorepository at the Stavanger University Hospital. Subsequent DNA and RNA retrieval and elaborate laboratory work outside routine diagnostics are performed at the Laboratory for Molecular Biology, except if otherwise stated.

All patient care are performed under the recommended national guidelines issued by the multidisciplinary Norwegian Gastrointestinal Cancer Group (NGICG), for both colorectal cancer (NGICG-CRC) and liver metastasis (NGICG-HPB), respectively.

### Collaboration

Interested collaborators are welcome to make contact. Discussion is extant with other groups and thus has the potential to generate an international cohort for comparison and validation of results. No external collaboration is yet confirmed but discussion in progress.

The cohort material will similarly also be available for cross-evaluation with other cohorts generated elsewhere, and will be beneficial for external validation purposes and hypothesis-generating experimental studies.

### Samples

Issues to attend to for biomarker research and reporting have been addressed in several leading journals [21, 22]. Consequently, the current study will seek to comply and report according to the biospecimen reporting for improved study quality (BRISQ) recommendations for all tissue sampling and storage in the study [44]. For parts of the study relating to clinical prognosis, we will aim to address the REMARK guidelines for biomarker research [45].

### Tissue samples

#### Formalin-fixed paraffin-embedded tumour/normal

Resected specimens are handled at the Department of Pathology according to protocol. An electronic template is followed and applied for gross examination and microscopic description of pathologic features and data for staging. Staging is done per the TNM-system (AJCC 7th edition). Representative tissue slides (resection ends or normal tissue distant from primary tumour; several tumour slides including most invasive front; all sampled lymph nodes) are formalin-fixed paraffin-embedded (FFPE) for routine H&E diagnostics and microscopy. Lymph nodes are sampled per protocol and aimed to achieve at least 12 nodes and, if less, a ‘lymph node revealing solution’ (a mixture composed of 95 % ethanol, diethyl ether, glacial acetic acid, and buffered formalin; also called GWEF) is applied to mesenteric fat in order to enhance node recovery [46].

#### Frozen fresh tumour tissue and normal sample

Before formalin fixation and immediate upon retrieval of the specimen, representative fresh tumour samples are obtained (at least three per tumour), stored in meticulously marked vials, and frozen in liquid nitrogen. Time is kept to a minimum between resection and delivery at Pathology in order to minimize loss of RNA quality [47, 48], usually delivered by an orderly within 15 min of retrieval from the operating room to the laboratory.

For the rare occasional procedures commencing or proceeding outside opening hours of the Department of Pathology, the surgeon in charge samples the fresh tumour biopsies per protocol and provides this in a portable insulated box container with dry ice for storage, normally <12 h. Samples are then collected by a technician and processed per protocol as early as possible the next morning.

### Blood samples

Peripheral blood samples are drawn (usually) from the antecubital vein on admission before surgery and on the outpatient follow-up appointment, approximately 4 weeks after surgery. Subsequent blood is drawn if patient is readmitted for new surgery for recurrence or metastatic disease, and then again if a second curative-intent surgery (e.g. resection of new large bowel tumour; local recurrence; or, metastasectomy) is planned. Blood samples are processed to serum and plasma by centrifugation and two vials of full blood (EDTA-containers) are frozen and stored in −80 °C freezers together with processed samples.

#### Circulating tumour cell (CTC) detection

Peripheral blood samples (9 ml) are collected in EDTA tubes and subjected to density centrifugation within 20 h (preferably 2 h) from the collection time. RNA is isolated from the peripheral blood mononuclear cell fraction and reverse transcribed. Circulating tumour cells (CTCs) are then detected indirectly by measuring epithelial-specific mRNAs, which are not present in normal blood cells, as surrogate markers [12, 49, 50]. mRNA concentrations are measured by quantitative reverse-transcription PCR. The background levels in blood samples from healthy control persons are utilized as a reference material to determine which patient samples are positive for CTCs.

### DNA/RNA extraction

DNA and RNA are extracted from freshly frozen tumour and normal (surgical resection margins or normal tissue sampled distant from primary tumour) using the QIACUBE (Qiagen) instrument and dedicated reagents and kits, according to manufacturers instructions. Weighted 15–20 mg of tissue are resuspended in lysis buffer and homogenized in the presence of 5 mm Ø steel beads, in a TissueLyser LT (Qiagen), at 50 Hz, for 4 min. Two consecutive protocols are then used on the QIACUBE instrument to extract DNA first, and RNA later (from flow through of first protocol) via the use of AllPrep DNA/RNA/miRNA Universal Kit. Concentration, purity (A260/280) and presence of phenol and protein contaminants in the eluted sample (A260/230) are measured and noted with a NanoDrop (ThermoFischer) intrument. Extracted DNA and RNA are labelled and stored at −80 °C in the aforementioned intramural biobanking facility.

### Employed molecular techniques

Mentioned examples here are not exclusive, but include:

#### Immunohistochemistry (IHC)

Antigen retrieval and antibody dilution are optimized prior to the study onset for the different antibodies. To ensure uniform handling of samples, all sections are processed simultaneously. Paraffin sections adjacent to the haematoxylin-eosin (H&E) sections used for histology are mounted onto Superfrost Plus slides and dried overnight at 37 °C followed by 1 h at 60 °C. Sections are deparaffinised in xylene and rehydrated in decreasing concentrations of alcohol. Antigen is retrieved using Tris–EDTA (pH 9.0) as the retrieval buffer. Endogenous peroxidase activity is blocked with a peroxidase-blocking reagent. The immune complex is visualized with the Dako REAL EnVision Detection System, Peroxidase/DAB, Rabbit/Mouse (K5007; Dako). Sections are incubated with EnVision/HRP, Rabbit/Mouse for 30 min and diaminobenzidine (DAB+) chromogen. The sections are counterstained with haematoxylin, dehydrated, and mounted. All steps are performed using DakoAutostainer and TBS (S1968; Dako) with 0.05 % Tween 20 as wash buffer. Quality assessment and scoring of the samples are executed by an experienced pathologist and with use of digital pathology software (Visiopharm) for some antibodies.

Candidate markers for investigation are in development, and includes (but not limited to) suggested markers for immune cells (e.g. CD4+, CD8+, CD45RO+) and as suggested in the Immunoscore [51], potential markers of differentiation (e.g. CDX2) in CRC stage subtypes [52], and markers related to mechanistic insight, such as MSH3 and its relation to EMAST [53, 54]. Other markers will be employed for specific subprojects as needed, e.g. for validating protein expression for gene variations.

#### Flowcytometry

Freshly drawn blood is collected in EDTA coated blood vacuum containers. Percentages of human CD4+ and CD8+ T-lymphocytes in erythrocyte-lysed whole blood are determined by flow cytometry. The antibody kit is acquired from BD Biosciences (Cat no: 342417) with CD3 FITC/CD8 PE/CD45 PerCP/CD4 APC conjugated staining. Cells are prepared according to protocol and 100 µl whole blood is used. BD Pharm Lyse (Cat no: 555899) is used at appropriate dilution to lyse erythrocytes. The samples are run on an Accuri C6 (BD biosciences) or a Cytoflex (Beckman-Coulter) flow cytometer both systems equipped with a blue and red laser, two light scatter detectors, and four fluorescence detectors with optical filters optimized for the detection of FITC, PE, PerCP, and APC. Further analysis of results is being done using the corresponding software (BD Biosciences Accuri C6 analysis software and CytExpert Beckman-Coulter).

#### Next generation sequencing (NGS)

Template preparation and chip loading is carried out using the Ion Chef™ System. With the use of CRC-specific, validated custom and commercially available panels, targeted DNA ion semiconductor sequencing of tumour material is performed on the Ion Torrent™ Personal Genome Machine^®^ (PGM, ThermoFischer) platform. Data analysis against the reference (human) genome is executed in-house with the aid of the Torrent Suite™ and Ion Reporter™ softwares.

### Statistical analysis and endpoints

The project is exploratory and thus no formal statistical power has been done. The population-based, observational, real-life, non-selected cohort will allow for adequate power based on the expected recruitment of a cohort size of 750 patients, of which an expected one-third (about 250) will have recurrence or death from disease. With the high number of events, this will allow for reasonable multivariable adjustments for outcomes. For smaller samples in subgroups (e.g. only stage II; or, only patients with resected liver metastasis), hypothesis-generating results will be pursued with appropriate sized prospective cohort samples with internal and external validation cohorts, where available.

The main endpoints will be cancer-specific, recurrence-free and overall survival, which will be analysed with Kaplan–Meier figures and log rank test. Exploratory analyses will be done using descriptive techniques for hypothesis-generating results. For laboratory values without established cut-offs, we will apply receiving-operator characteristics (ROC) analyses for optimal cut-off determination [55]. For prognostic factors, we will apply appropriate multivariable regression analyses for appropriately adjusted analyses.

## Discussion

The ACROBATICC projects aim to integrate the routine clinical work-up and treatment of patients with primary CRC and resectable liver metastasis with state-of-the-art molecular technology investigations of blood samples and tumor tissues. The aim is to explore and identify better predictive and prognostic biomarkers that may eventually help in clinical decision-making for more precise, personalized and tailored treatment. The ACROBATICC clinical and molecular biobank repository will serve as a long-term source for novel exploratory analysis and invite collaborators for mutual validation of promising biomarker results. The project aims to generate results that can help better discern prognostic groups in stage II/III cancers; explore prognostic and predictive biomarkers, and help detail the biology of colorectal liver metastasis for better patient selection and tailored treatment [23, 28, 56].

The role of population-based cancer biobanking is increasingly recognized as important for exploratory and confirmatory studies at an unselected, population-level. While regular diagnostic biobanking [i.e. formalin-fixed and paraffin embedded (FFPE) tissue blocks] allow for valuable analyses outside routine descriptive data, such repositories may have a number of medicolegal and laboratory limitations which may be overcome by specific research-driven projects. The current translational cancer research project will allow for further in-depth analyses into cancer biology otherwise not available from material obtained by routine care.

The use of “liquid biopsy” has gained considerable attention as a novel source of biomarkers. Blood-based biomarkers could prove to be practical tools for CRC detection, as the monitoring of biomarkers in biological fluids offers many advantages, including minimal invasiveness and easy accessibility [57]. In the current study, we will have the opportunity to explore for tumour-specific markers in blood and tissue that may be related to prediction and prognosis of outcome.

Lack of uniform research designs, poor quality control and large variation in reporting have hampered biomarker research and comparison of data in the past. This has invariably led to a number of promising but non-validated biomarkers in past studies. Currently, a large number of guidelines and recommendations are available to instruct, inform and impede better and more uniform reporting of results. However, the number of such guidelines is increasing rapidly [58], with some suggesting there be too many guidelines to possibly comply to. However, we believe that a core set of important guides help set useful framework for reporting and help avoid huge deviation from recommended practice. Evaluation of compliance to such guidelines suggest that considerable deviation and lack of reporting core data still exist in biomarker research studies [59]. Thus, we would seek to adhere and comply with the recommendations addressed in the protocol and any other relevant recommendations, as issued by the EQUATOR network (http://www.equator-network.org).

### Project status

The project is currently recruiting patients and laboratory work on the CTCs is ongoing, as well as laboratory work on MSI and EMAST in primary tumours. Hot spot panels of known CRC mutations with NGS technology is being prepared. A pilot, feasibility study to test for same-time comparison of patients’ circulating immune-cells in peripheral blood and comparison to tumour-infiltrating cells in the cancer specimen is currently being conducted.

### Future aspects

We envision several add-ons to be possible with increases resources and manpower in the project. For one, patient reported outcomes (PROs) is an increasing are of interest and would yield yet another dimension to the clinical-translational aspect of the project [60]. Also, the sampled biopsies will allow for a number of other experiments and analyses, such as exosomic DNA, microRNA and other emerging biomarkers. Further, other sampling techniques and specimens would be feasible in the future, such as sampling and investigating faeces for both genetic and epigenetic biomarkers [24], but also investigating the microbiome for its putative role in carcinogenesis but also possible influence on cancer biology [61, 62]. Last, but not least, we would pursue international collaboration for mutual validation of similar ongoing biomarker projects [63]. The prospective cohort results will seek collaboration for external validation studies but may also serve as an external validation cohort for other research groups interested in collaboration.

## Abbreviations

CRC: colorectal cancer
CLM: colorectal liver metastasis
CTC: circulating tumour cell
NGS: next-generation sequencing
EMAST: elevated microsatellite alterations in selected tetranucleotides
MSI: microsatellite instability
IHC: immunohistochemistry
TNM: tumour-node-metastasis
CIMP: CpG-island methylator phenotype
CIN: chromosomal instability
SUH: Stavanger University Hospital
NGICG: Norwegian Gastrointestinal Cancer Group
FFPE: formalin-fixed paraffin-embedded
GWEF: glacial acetic acid, water, ethanol, formalin
ROC: receiving-operator characteristics
H&E: haematoxylin and eosin
PROs: patient reported outcomes
